# Versatile Solid Modifications of Multicomponent Pharmaceutical Salts: Novel Metformin–Rhein Salts Based on Advantage Complementary Strategy Design

**DOI:** 10.3390/pharmaceutics15041196

**Published:** 2023-04-09

**Authors:** Mingchao Yu, Meidai Liang, Qi An, Wenwen Wang, Baoxi Zhang, Shiying Yang, Jian Zhou, Xiuying Yang, Dezhi Yang, Li Zhang, Guanhua Du, Yang Lu

**Affiliations:** 1 Institute of Materia Medica, Peking Union Medical College, Chinese Academy of Medical Sciences, Beijing 100050, China; 2 Beijing Key Laboratory of Polymorphic Drugs, Center of Pharmaceutical Polymorphs, Beijing 100050, China; 3 Beijing Key Laboratory of Drug Targets Identification and Drug Screening, Beijing 100050, China

**Keywords:** drug–drug salt, polymorphism, solubility, hygroscopicity, hypoglycemic activity, diabetic complications

## Abstract

This study aimed to develop an effective treatment for diabetes and diabetic complications, based on the advantage complementary strategy of drug–drug salt, by designing and synthesizing the multicomponent molecular salts containing metformin (MET) and rhein (RHE). Finally, the salts of MET–RHE (1:1), MET–RHE–H_2_O (1:1:1), MET–RHE–ethanol–H_2_O (1:1:1:1), and MET–RHE–acetonitrile (2:2:1) were obtained, indicating the polymorphism of salts formed by MET and RHE. The structures were analyzed by the combination of characterization experiments and theoretical calculation, and the formation mechanism of polymorphism was discussed. The obtained results of in vitro evaluation showed that MET–RHE had a similar hygroscopicity with metformin hydrochloride (MET·HCl), and the solubility of the component of RHE increased by approximately 93 times, which laid a foundation for improving the bioavailability of MET and RHE in vivo. The evaluation of hypoglycemic activity in mice (C57BL/6N) indicated that MET–RHE exhibited better hypoglycemic activity than the parent drugs and the physical mixtures of MET and RHE. The above findings demonstrate that this study achieved the complementary advantages of MET and RHE through the multicomponent pharmaceutical salification technique, and provides new possibilities for the treatment of diabetic complications.

## 1. Introduction

According to the statistics of the International Diabetes Federation in 2017, the prevalence of diabetes in the world is gradually increasing, and more than 90% of patients with diabetes mellitus experience Type 2 diabetes (T2D) [1]. Considering the complex pathogenesis and complications of diabetes, most patients with T2D cannot achieve the glycemic targets with monotherapy, and combination therapy is usually used to achieve glycemic control. However, the complex dosing regimens pose the challenge of reducing patient compliance [2]. Based on these dilemmas, the fixed-dose combination (FDC) therapy provides a way to simplify complex treatment regimens [3]. In addition, FDC reduces adverse reactions, reduces economic costs, and improves medication concordance [2]. Notably, the stability and solubility differences between the components of FDC still limit its clinical application [4]. Therefore, an appropriate method needs to be developed to solve the problem of clinical application of multicomponent drugs. In recent years, the application of multicomponent pharmaceutical cocrystals/salts in the field of drug combination has shown unique advantages, thus improving the physicochemical properties of active pharmaceutical ingredients (APIs) and showing the potential to reduce side effects and improve pharmacological activities [5,6,7,8,9,10].

**Figure 1:**
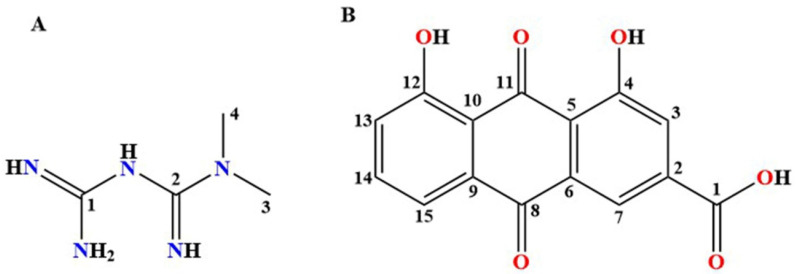
Chemical structures of metformin (**A**) and rhein (**B**).

Metformin (MET, pK_a_ = 12.4 [11], Figure 1A), as a biguanide hypoglycemic agent, is the first choice for the treatment of T2D [12], especially for obese patients [13]. With the deepening of the research on MET, researchers have found that MET has unique pharmacological activity in the treatment of cancer [14,15,16], aging [17], Alzheimer’s disease [18,19], cardiovascular diseases [20], polycystic ovary syndrome [21], obesity [22,23], diabetic nephropathy [24,25,26], and other diseases [27]. The drawback is that while metformin has excellent solubility, it encounters the problem of high moisture absorption, thus introducing strict environmental requirements for the production, storage, and transportation of MET [28]. Based on this dilemma, MET is commercially available, usually in the form of metformin hydrochloride, to reduce its hygroscopicity [29]. At the same time, the large amount of chloride ion introduced in the form of hydrochloride has no pharmacological activity, but may have the same ion effect as the chloride ion present in the stomach. Based on clinical studies, nausea, vomiting, diarrhea, and other gastrointestinal side effects occur in 20–30% of patients taking metformin hydrochloride [30]. Considering the above problems, researchers have carried out multicomponent co-crystallization studies on MET via salification strategy [6,11,31,32,33,34,35,36,37,38,39,40,41,42]. However, these literature reports mainly focused on the optimization of MET physicochemical properties, and no pharmacological evaluation has been conducted on the hypoglycemic effect of multicomponent salts in vivo.

Rhein (RHE, pKa = 3.2, Figure 1B) is an anthraquinone compound extracted from Polygonaceae plants such as rhubarb and Polygonum multiflorum [43,44]. RHE has various pharmacological activities, including hypoglycemic [45], renal function regulation [46], antitumor [47], anti-inflammatory [48,49], and antibacterial [50]. Notably, RHE has unique potential for the treatment of diabetes nephropathy (diabetic complications) [51] because of its hypoglycemic and lipid-lowering activity [45], islet cell protection activity [52,53], anti-inflammatory activity [48,49,54], and renal function regulation activity [51,55,56]. According to the Biopharmaceutics Classification System (BCS) classification, RHE belongs to the BCS II class of drugs, with superior permeability and unsatisfactory solubility [57]. The unacceptably low water solubility of RHE causes the problem of low bioavailability, which greatly limits the clinical application of RHE [58]. Considering the unique advantages of multicomponent pharmaceutical salts in improving the physicochemical properties of APIs [59], the method of salification was adopted to improve the solubility of RHE.

First, according to the ΔpK_a_ rule [60], the possibility of the multicomponent salt formation was predicted. The ΔpK_a_ of MET (pK_a_ = 12.4) and RHE (pK_a_ = 3.2) were greater than 3, allowing the prediction of the potential for multicomponent salt formation between MET and RHE [60]. Moreover, from the structural point of view, MET and RHE are rich in hydrogen bond donors and hydrogen bond acceptors, and it is feasible to form hydrogen bonds between them. Pharmacologically, both MET and RHE have hypoglycemic [12,13,45] and diabetic nephropathy-relieving activities [24,25,46,51,56,61], which lays a foundation for enhancing the activity of MET–RHE multicomponent salts in controlling diabetes and diabetes complications. In addition, the gastrointestinal side effects of MET can be reduced by delaying its sustained release in vivo [62], while the low-solubility of RHE contributes to the sustained release of MET, which will help in reducing the gastrointestinal side effects of MET to patients, and is expected to reduce the hygroscopicity of MET at the same time [6,42]. From another point of view, by preparing dual-drug molecular salt with highly soluble MET, the low solubility of RHE can be improved, thereby increasing the bioavailability of RHE. The above design idea gives full play to the concept of complementary advantages of multicomponent pharmaceutical cocrystals.

Based on the above salification strategy, four multicomponent pharmaceutical salts and their solvates were obtained, including MET–RHE (1:1), MET–RHE–H_2_O (1:1:1), MET–RHE–ethanol–H_2_O (1:1:1:1), and MET–RHE–acetonitrile (2:2:1). The prepared multicomponent pharmaceutical salts were characterized by single-crystal X-ray diffraction analysis (SXRD), powder X-ray diffraction analysis (PXRD), and thermal analysis (DSC-TGA). On the basis of mastering the processes of crystal formation and transformation from the solvates to MET–RHE, the stability, hygroscopicity, and solubility of MET–RHE in vitro and its hypoglycemic activity in mice were evaluated systematically. The experimental results of this thesis showed that MET–RHE not only optimized the solubility of RHE and reduced the hygroscopicity of MET, but also ameliorated the hypoglycemic activity in mice, thus providing a material basis for the further synergistic effects of MET and RHE to alleviate diabetic nephropathy.

This study shows that the multicomponent pharmaceutical salt based on the strategy of complementary advantages can improve the physicochemical properties shortcomings of the single component and synergistically enhance the efficacy, which provides a new way to improve the bioavailability of the original drugs in vivo and to maximize their multiple potential pharmacological activities to a greater extent.

## 2. Materials and Methods

### 2.1. Compounds and Agents

MET·HCl (purity > 99%, MW = 166) and RHE (purity > 99%, MW = 284) were purchased from Nine-Dinn Chemistry Co., Ltd. (Shanghai, China) and Hubei Wande Chemical Co., Ltd. (Tianmen, China), respectively. The reagents used were of analytical grade. The solid drug delivery device (patent number: 201010219220.5) was developed and prepared by the National Drug Screening Center of the Institute of Materia Medica, Chinese Academy of Medical Sciences.

### 2.2. Preparation of MET Base

Metformin hydrochloride was dehydrochlorinated by acid–base neutralization method, and the final metformin sample was obtained. First, 3.31 g (20 mmol) of metformin was accurately weighed and dissolved in an appropriate amount of isopropanol, to which 0.80 g (20 mmol) of sodium hydroxide was added. The mixture was stirred at 300 rpm at room temperature overnight. The solution was filtrated, and the product was spun dry to obtain the metformin sample for the experiment at 60 °C and −0.01 MPa.

### 2.3. Preparation of Multicomponent Salts

For MET–RHE (1:1), 0.2 mmol of MET (25.8 mg) and 0.2 mmol of RHE (56.8 mg) were accurately weighed into a mortar, 2 mL of ethanol was added, and the solid was manually grounded at 25 °C for 1 h. The solid was dried at 25 °C for 4 h to obtain an auratus powder sample. The obtained sample was dissolved in 6 mL of methanol, and then filtered. The filtrate was air-dried at 25 °C, and faint yellow massive crystals were obtained after 6 days.

For MET–RHE–H_2_O (1:1:1), 0.5 mmol of MET (64.5 mg) and 0.5 mmol of RHE (142.0 mg) were accurately weighed into a vial, 5 mL of acetone/water (*v*/*v*, 1:1) mixed solvent was added, and the mixture was stirred at 25 °C at 300 rpm for 3 h. The solvent was quickly removed using a rotary evaporator, and the resulting solid was dried at 50 °C for 2 h.

For MET–RHE–ethanol–H_2_O (1:1:1:1), 0.1 mmol of MET (12.9 mg) and 0.1 mmol of RHE (28.4 mg) were accurately weighed into a mortar, and 3 mL of ethanol/water (*v*/*v*, 1:1) mixed solvent was added and ground manually at 25 °C for 2 h. A brown powder was finally obtained. The resulting sample was dissolved in 8 mL of ethanol and filtered. The filtrate was air-dried at 25 °C. After 5 days, yellow massive crystals suitable for single-crystal X-ray diffraction were obtained.

For MET–RHE–acetonitrile (2:2:1), 0.5 mmol of MET (64.5 mg) and 0.5 mmol of RHE (142.0 mg) were accurately weighted into a vial, and added to 12 mL of methanol/acetonitrile (*v*/*v*, 3:1) mixed solvent. The mixture was stirred at 25 °C at 300 rpm speed for 48 h, and then filtered. The filtrate was air-dried at 25 °C, and tangerine block-like crystals were obtained after 7 days.

### 2.4. Powder X-ray Diffraction (PXRD)

PXRD experiments were performed using a Rigaku D/max-2550 diffractometer (Rigaku, Tokyo, Japan), which was equipped with a Cu–Kα radiation source set at 40 kV and 150 mA. In this paper, the diffraction data were collected in the 2θ range of 3–80° with a scan rate of 8°/min. All the data were analyzed using Jade 6.0 software.

### 2.5. Thermal Analysis

DSC characterization of the experimental samples were performed in this study using a Mettler Toledo DSC/DSC 1 (Mettler Toledo, Greifensee, Switzerland). The specific method is to accurately weigh the sample into an alumina crucible, start heating from 30 °C, and complete the whole experiment at a heating rate of 10 °C min^−1^ with a flow of 50 mL/min of N_2_.

The TGA analysis for this study was performed by a Mettler Toledo DSC/TGA1 (Mettler Toledo, Greifensee, Switzerland). An appropriate amount of sample was weighed in a crucible using 40 µL alumina and warmed to 500 °C at 10 °C/min starting at 30 °C under a N_2_ flow of 50 mL/min.

The STAR software package (STARe Default DB V9.10, Mettler Toledo, Greifensee, Switzerland) was used for the processing and analysis of DSC and TGA results in this experiment.

### 2.6. Single-Crystal X-ray Diffraction (SXRD)

SCXRD was carried out on a Rigaku MicroMax-002+ diffractometer (Rigaku Americas, The Woodlands, TX, USA) with Cu–Kα radiation (λ = 1.54178 Å) at the temperature of 293 K. The structures of qualified single crystal samples obtained in this study were solved by direct method and refined with the full-matrix least-squares technique. The nonhydrogen atoms were refined using anisotropic displacement parameters, and hydrogen atoms were placed at the calculated positions and refined using a riding model.

### 2.7. Theoretical Computation

The counterpoise-corrected interaction energies in the salts were investigated by density functional theory using the Gaussian 16 program [63]. The geometries of all the hydrogen atoms were optimized at the B3LYP-D3/6-311+G (d, p) level, and the original X-ray coordinates of all the heavy atoms were maintained. Then, the single-point energies were calculated at the M06-2X/def2-TZVP level [64]. The Multiwfn 3.8 software [65] was employed for the analysis of molecular planarity. The CrystalExplorer 21 program was used to calculate the lattice energy [66].

### 2.8. Crystal Form Transformation Analysis

In order to investigate the transformation process from the solvates to MET–RHE salt, the MET–RHE solvates were desolvated at a temperature of at least 20 °C above the corresponding solvent peak temperature in DSC curves. The products were analyzed by DSC and PXRD in comparison with the MET–RHE salt, respectively.

### 2.9. Stability Study

To investigate the stability of MET and MET–RHE, powder samples were placed in high-temperature (60 °C), high-humidity (25 °C, 90 ± 5%), and illuminated (4500 lx ± 500 lx) environment for 10 days. The stability of samples was evaluated via PXRD analysis.

### 2.10. Hygroscopicity Study

The hygroscopicity of MET and MET–RHE was studied by dynamic vapor sorption experiment (DVS Adventure, Surface Measurement Systems, London, United Kingdom.). The samples were studied at 25 °C in the humidity range of 20–95% relative humidity (RH). Each humidity step was performed when a change in weight of less than 0.02% occurred within 10 min, with a maximum retention time of 120 min.

### 2.11. Powder Dissolution and Solubility In Vitro

Prior to the in vitro dissolution and solubility study, the samples were processed with a 100-mesh sieve in advance to minimize the influence of sample particle size on the experimental results. In addition, the aqueous equilibrium solubilities of ionizable drugs are measured in buffered solutions, considering that their solubilities are a pH-dependent parameter [67], and the Britton–Robinson buffer at pH = 7 was used as medium during the experiment [67,68].

Afterward, precisely weighed RHE (30.0 mg) and MET–RHE (equivalent to 30.0 mg RHE) were added to a dissolution vessel containing 450 mL of buffer medium (pH = 7). Samples were stirred at 100 rpm at 37 °C, and 1 mL of sample was obtained at 0, 5, 15, 30, 50, 75, 105, 150, 210, 270 and 390 min, while the same volume of buffer was replenished. The samples were analyzed for dissolved concentration by HPLC (Agilent 1200, Agilent Technologies Inc., Palo Alto, CA, USA) after filtration through 0.22 µm PTFE filter. The liquid-phase conditions were as follows: Welch Ultimate AQ-C18 (250 mm × 4.6 mm, 5 μm); mobile phase, methanol–0.2% formic acid (85:15, *v*/*v*); detection wavelength, 254 nm; flow rate, 1.0 mL/min; column temperature, 30 °C; injection volume, 10 μL. The experiment was repeated three times and the average value was taken.

The solubility of MET–RHE and RHE was studied using the saturation shake-flask method [67]. Approximately 100 mg of sample and a rotor were added to a tube containing 10 mL of buffer medium (pH = 7) and then capped and stirred at 37 °C for 48 h at 200 rpm. The precipitate was allowed to settle, and after filtration through a 0.22 µm polytetrafluoroethylene (PTFE) filter, the sample was analyzed for dissolved concentration by HPLC (Agilent 1200). The liquid-phase conditions were the same as above. The experiment was repeated thrice, and the average value was obtained. The remaining solid was dried and characterized by PXRD.

### 2.12. In Vivo Hypoglycemic Activity Evaluation

Male C57BL/6 mice (7 weeks old) purchased from Vitaliver (Beijing, China) were used in this study and placed under environmentally controlled conditions with a light/dark cycle for 12 h at a temperature of 22 ± 3 °C and a humidity of 55 ± 5%. Mice were injected intraperitoneally (i.p.) with 120 mg/kg streptozotocin (STZ, InnoChem Science & Technology Co., Ltd., Beijing, China) after fasting for 16 h to induce diabetic models. The control animals received an i.p. injection of an equal volume of vehicle only (citrate buffer, pH 4.5). Seven days after the administration of STZ, the 4 h fasting blood glucose levels of mice were measured with an ACCU-CHEK^®^ Active glucometer (Roche, Hoffmann, Germany). Mice with fasting glucose level over 11 mmol/L were selected as diabetic mice models. In addition to the normal control group, the diabetic mice were divided into five groups, including the diabetic model group, MET·HCl group (100 mg/kg, 0.6 mmol/kg, M = 165.6 g/mol), MET–RHE group (249.6 mg/kg, 0.6 mmol/kg, M = 413.4 g/mol), physical mixture of MET and RHE group (271.6 mg/kg, 0.6 mmol/kg MET·HCl + 0.6 mmol/kg RHE), and RHE group (171.6 mg/kg, 0.6 mmol/kg, M = 284.2 g/mol). All drug dosages were converted to 0.6 mmol/kg. The mice from different administration groups were administered with drugs by gavage after 4 h of fasting. Normal control and diabetic model groups were filled with equal doses of normal saline. Blood glucose levels were measured at 0, 1, 2, 4, 6 and 8 h after administration.

## 3. Results and Discussion

### 3.1. PXRD

**Figure 2:**
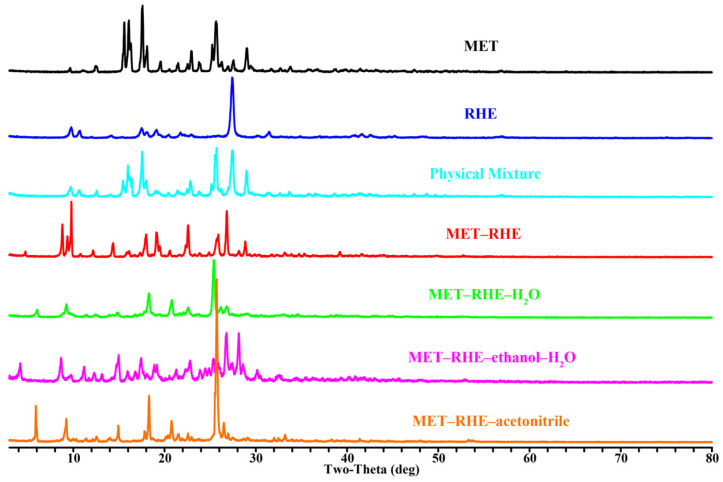
PXRD comparisons of MET, RHE, physical mixture, MET–RHE, MET–RHE–H_2_O, MET–RHE–ethanol–H_2_O, and MET–RHE–acetonitrile.

As a convenient and effective characterization means for solid-state crystalline substances [69], PXRD was adopted to further identify the formation of the multicomponent salts, such as MET, RHE, physical mixture (molar ratio of MET to RHE = 1:1), MET–RHE, MET–RHE–H_2_O, MET–RHE–ethanol–H_2_O, and MET–RHE–acetonitrile (Figure 2). The characteristic diffraction peaks were observed at 2θ values of 12.48°, 15.60°, 16.10°, 16.32°, 17.60°, 18.10°, 19.60°, 21.50°, 22.53°, 22.96°, 23.80°, 25.24°, 25.66°, 27.00°, 27.58° and 29.66° for MET and 9.78°, 10.74°, 17.50°, 18.04°, 19.14°, 21.76°, 27.42° and 31.50° for RHE. The intensity and position of the characteristic diffraction peaks in the PXRD spectra of the four multicomponent salts exhibited remarkable difference compared to MET and RHE, as indicated by both the disappearance of old peaks and the generation of new peaks, which were considerably different from the simple physical mixture of MET and RHE, indicating that four new phases different from the two raw ingredients were synthesized in the experiment [70].

### 3.2. Thermal Analysis

**Figure 3:**
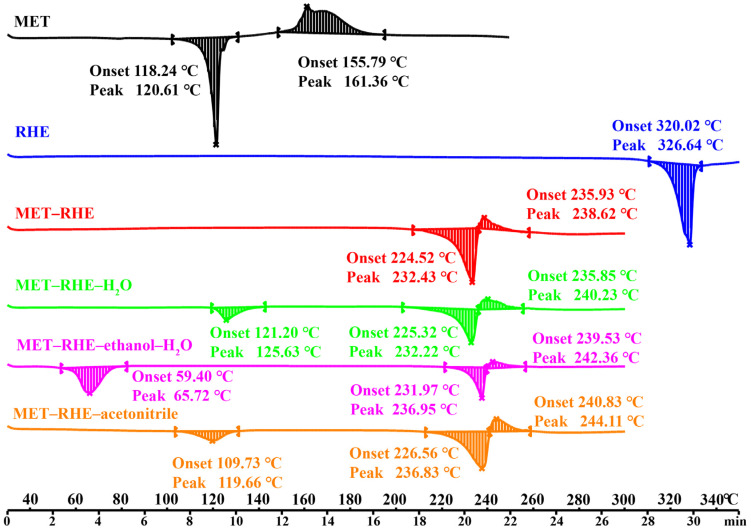
DSC profiles of MET, RHE, MET–RHE, MET–RHE–H_2_O, MET–RHE–ethanol–H_2_O, and MET–RHE–acetonitrile.

As a sensitive thermal analysis technique, DSC can be used to analyze the formation and purity of new phases. The DSC results for MET, RHE, and multicomponent salts are provided in Figure 3. MET has an endothermic peak around 121 °C and a resolving exothermic peak around 161 °C, while RHE only has an endothermic peak at 327 °C. By contrast, a sharp endothermic peak of MET–RHE appeared at approximately 232 °C, followed by the thermal decomposition at approximately 239°, which is obviously different from the melting point of MET and RHE, and no obvious endothermic peak was observed at low temperature, indicating that MET–RHE is a new phase different from MET and RHE and does not contain solvent. Notably, unlike MET–RHE, MET–RHE–H_2_O, MET–RHE–ethanol–H_2_O, and MET–RHE–acetonitrile also have endothermic peaks at about 126, 66, and 120 °C, respectively, and the endothermic peaks of MET–RHE–H_2_O and MET–RHE–acetonitrile are close to that MET (121 °C). However, the PXRD pattern of MET–RHE–H_2_O and MET–RHE–acetonitrile has no characteristic peak that can prove the remaining MET. Therefore, the endothermic peaks at low temperatures of MET–RHE–H_2_O, MET–RHE–ethanol–H_2_O, and MET–RHE–acetonitrile correspond to the presence of solvents in their structures.

Moreover, the thermal behavior also appears with the change of the TG profiles of the multicomponent salts. The TGA profile of the multicomponent pharmaceutical salt is shown in Figure S1. No mass loss was observed in the test temperature range below the melting point of MET–RHE, indicating that no solvent molecules were present in the lattice of MET–RHE. The solvent weight loss temperatures of the TG curves of MET–RHE–H_2_O, MET–RHE–ethanol–H_2_O, and MET–RHE–acetonitrile correspond to the solvent endothermic peaks of their DSC curves, while the percentage of solvent weight loss steps is quite close to the theoretical percentage (Table S1), which is consistent with the following SXRD resolution results. This finding indicates the high purity of the prepared powder samples.

### 3.3. SXRD

Three multicomponent pharmaceutical salts were obtained, including MET–RHE, MET–RHE–ethanol–H_2_O, and MET–RHE–acetonitrile, and their crystallographic data were deposited in the Cambridge Crystallographic Data Center. The crystal data and structure refinement parameters of the three multicomponent pharmaceutical salts are presented in Table 1.

**Figure 4:**
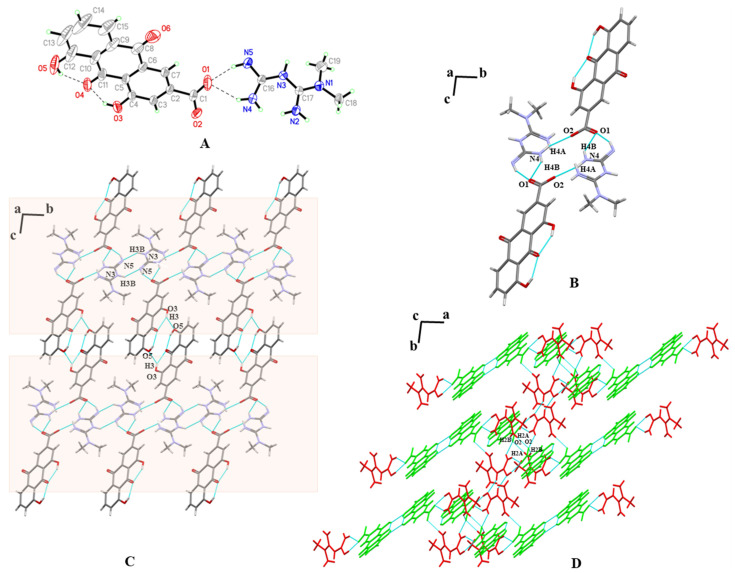
(**A**) An asymmetric unit of MET–RHE. (**B**) The tetrameric ring motif $R_{4}^{4}(12)$. (**C**) The two-dimensional structure of MET–RHE. (**D**) The three-dimensional structure of MET–RHE (red: MET, green: RHE).

MET–RHE: Single-crystal X-ray diffraction reveals MET–RHE crystallized in the triclinic *P-1* space group. According to Figure 4A, it can be judged that a proton transfer occurred between MET and RHE, and an asymmetric unit consists of one MET cation and one RHE anion. MET is connected to RHE by N_5_–H_5A_···O_1_ and N_4_–H_4B_···O_1_ hydrogen bonds, in addition to the presence of O_5_–H_5_···O_4_ and O_3_–H_3_···O_4_ intramolecular hydrogen bonds in RHE. Detailed hydrogen bonding information is listed in Table S2, and more crystallographic data can be viewed at CCDC 2241616. From the a-axis perspective, two adjacent asymmetric units form a centrosymmetric tetrameric ring through N_4_–H_4A_···O_2_ hydrogen bonding, described as $R_{4}^{4}\left(12\right)$ in graph set notation (Figure 4B). The abovementioned centrosymmetric tetrameric rings are connected by N_3_–H_3B_···N_5_ hydrogen bonds between adjacent METs to form a chain-like structure extending indefinitely along the b-axis. The chains are connected to each other by O_3_–H_3_···O_5_ hydrogen bonds between adjacent RHEs, forming a planar structure that extends wirelessly along the c-axis direction (Figure 4C). On the basis of the abovementioned planar structure, the planes are stacked layer by layer via N_2_–H_2A_···O_2_ and N_2_–H_2B_···O_2_ hydrogen bonds, the π···π stacking, and other weak interactions, forming a three-dimensional structure, as shown in Figure 4D.

**Figure 5:**
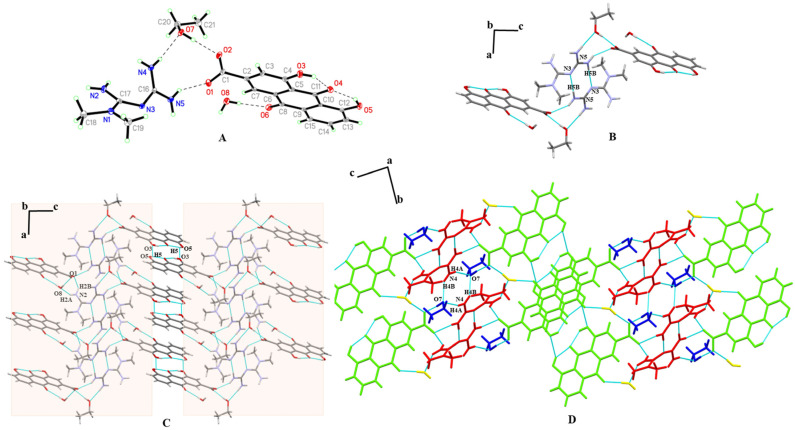
(**A**) An asymmetric unit of MET–RHE–ethanol–H_2_O. (**B**) The dimeric ring motif $R_{2}^{2}(8)$. (**C**) The two-dimensional structure of MET–RHE–ethanol–H_2_O. (**D**) The three-dimensional structure of MET–RHE–ethanol–H_2_O (red: MET, green: RHE, blue: ethanol, yellow: H_2_O).

MET–RHE–ethanol–H_2_O: An asymmetric unit of MET–RHE–ethanol–H_2_O contains one MET cation, one RHE anion, one ethanol molecule, and one water molecule (Figure 5A). It is clearly seen that MET cation, RHE anion, and ethanol molecules through N_4_–H_4A_···O_7_, N_5_–H_5A_···O_1,_ and O_7_–H_7A_···O_2_ hydrogen bonding. RHE molecules are linked to water molecules through O_8_–H_8A_···O_6_ hydrogen bonds while forming intramolecular hydrogen bonds through O_3_–H_3_···O_4_ and O_5_–H_5_···O_6_ hydrogen bonds. Detailed hydrogen bonding information is listed in Table S3, and more crystallographic data can be viewed at CCDC 2241591. Similar to the single crystal structure of MET–RHE, from the perspective of the b-axis, the two asymmetric units of MET–RHE–ethanol–H_2_O form a centrosymmetric dimer via N_5_–H_5B_···N_3_ hydrogen bonds, depicted in the atlas notation as $R_{2}^{2}(8)$ (Figure 5B). The dimers are linked by N_2_–H_2A_···O_8_ and N_2_–H_2B_···O_1_ hydrogen bonds, resulting in a chain-like structure that extends infinitely along the a-axis. The chains are linked with each other by the dimeric structure formed by O_5_–H_5_···O_3_ hydrogen bonds between RHEs, which subsequently forms a planar structure extending infinitely along the c-axis (Figure 5C). On the basis of the above planar structure, the planes are stacked layer by layer through N_4_–H_4A_···O_7_ and N_4_–H_4B_···O_7_ hydrogen bonds, π···π stacking, and other weak interactions to finally form a three-dimensional structure, as shown in Figure 5D.

**Figure 6:**
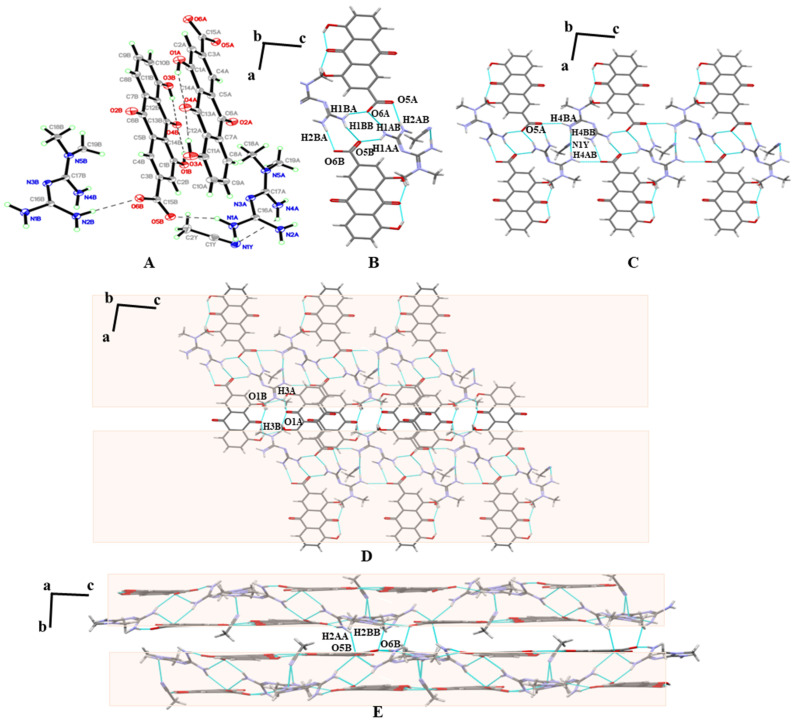
(**A**) An asymmetric unit of MET–RHE–acetonitrile. (**B**) The tetrameric ring motif $R_{4}^{4}(20)$. (**C**) A one-dimensional chain along the b-axis. (**D**) The two-dimensional structure of MET–RHE–acetonitrile. (**E**) The three-dimensional structure of MET–RHE–acetonitrile.

MET–RHE–acetonitrile: An asymmetric unit of MET–RHE–acetonitrile contains two MET cations, two RHE anions, and one acetonitrile molecule. The RHEs still have intramolecular hydrogen bonds in the lattice and are connected with the MET molecule through N_2B_–H_2BB_···O_6B_ and N_1A_-H_1AA_···O_5B_ hydrogen bond, while the acetonitrile molecule is connected with the MET molecule through N_1Y_–H_4AB_···N_4A_ hydrogen bond (Figure 6A). Detailed hydrogen bonding information is listed in Table S4, and more crystallographic data can be viewed at CCDC 2241615. By analyzing the bonding mode of single crystals, we were surprised to find that the single crystal structure of MET–RHE–acetonitrile is obviously different from that of MET–RHE and MET–RHE–ethanol–H_2_O. In the structure of MET–RHE–acetonitrile, two MET cations and two RHE anions are linked by N_2B_–H_2BA_···O_6B_, N_1B_–H_1BB_···O_5B_, N_1B_–H_1BA_···O_6A_, N_1A_–H_1AB_···O_6A_, N_2A_–H_2AB_···O_5A_, and bN_1A_–H_1AA_····O_5B_ hydrogen bonds to form a tetrameric ring (Figure 6B). The tetrameric rings are connected by N_4B_–H_4BA_····O_5A_, N_4B_–H_4BB_····N_1Y_, and N_4A_–H_4AB_···N_1Y_ hydrogen bonds to form a chain-like structure extending infinitely along the c-axis (Figure 6C). The chains are further linked by O_3A_–H_3A_···O_1B_ and O_3B_–H_3B_····O_1A_ hydrogen bonds and extend infinitely along the a-axis (Figure 6D). Finally, the layers are connected by N_2A_–H_2AA_···O_5B_ and N_2B_–H_2BB_···O_6B_ hydrogen bonds, π···π stacking, and other weak interactions to form a three-dimensional structure, as shown in Figure 6E, which is not a simple sandwich layer array.

### 3.4. Theoretical Computation

#### 3.4.1. The Interaction Energies and Lattice Energy Analysis

MET and RHE molecules were interacted in different H-bond motif in the salts. The interaction energy was analyzed by theoretical calculation, and the interaction energies in different types are listed in Table 2. Among these motifs, the $R_{2}^{2}(8)$ H-bond motif was found only in MET–RHE–acetonitrile salt, and its interaction energy was strongest (−96.79 kcal/mol), which led to its higher lattice energy.

The acetonitrile molecule in MET–RHE–acetonitrile has no classical hydrogen bond interaction with other molecules, but its desolvation peak temperature in the DSC curve is higher than that of MET–RHE–ethanol–H_2_O, which is speculated to be caused by its higher lattice energy. In this paper, Crystal Explorer software is used to calculate the lattice energy of the two complexes. Although this method is not suitable for salt systems, only qualitative comparison is made, and the calculation results of this case are still able to illustrate the problem. The parameter of radius of 20 Å was selected to calculate the lattice energy with the default setting. The lattice energy was −101.02 and −530.04 kcal/mol for MET–RHE–ethanol–H_2_O and MET–RHE–acetonitrile, respectively. This result reasonably supports our previous speculation.

#### 3.4.2. The Molecular Planarity Analysis

The RHE dimer structures existed in all these salts, but, interestingly, slight differences in the planarity of the dimers were observed, which influenced the strength of the interaction of RHE molecules in the dimers. The planarity of the dimer was characterized by molecular planarity parameter (MPP) and span of deviation from plane (SDP) parameters, and the results are listed in Table 3. MPP reflects the overall planarity of the area under consideration, while SDP measures the maximum deviation from the fitted plane in the area under consideration, that is, the maximum span perpendicular to the fitted plane. The results show that the order of MPP values was consistent with the interaction energies of the corresponding H-bond motifs, that is, the MET–RHE– acetonitrile dimer with the best planarity has the strongest interaction, and the MET–RHE– ethanol–H_2_O interaction has the lowest interaction energy. However, the order of MPP values was inconsistent with the hydrogen bond length, so the effect of molecular planarity is greater than that of hydrogen bond length for the strength of interactions.

### 3.5. Crystal Form Transformation Analysis

**Figure 7:**
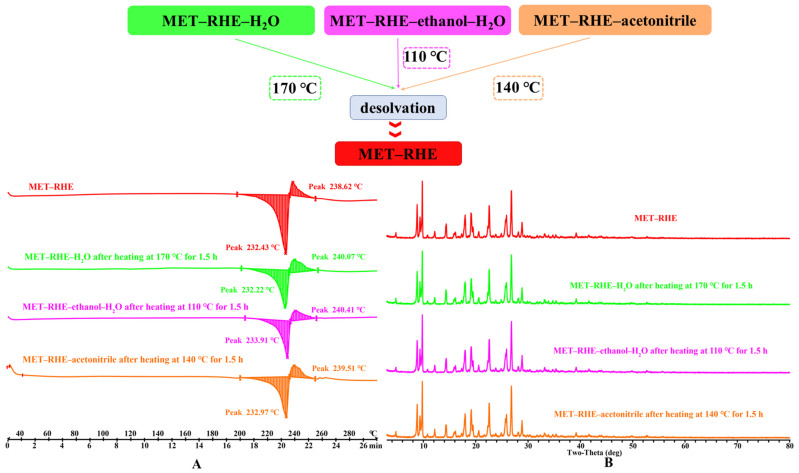
(**A**) Comparison of DSC profiles after desolvation. (**B**) Comparison of PXRD profiles after desolvation.

The presence of solvent molecules in the cocrystal can lead to the presence of additional chemical entities in the drug substance during the development process. In the present study, the temperature required for solvent removal was determined by DSC, and the direction of the crystal transformation process was determined based on the PXRD pattern. According to the DSC curve of MET–RHE–H_2_O, MET–RHE–ethanol–H_2_O, and MET–RHE–acetonitrile (Figure 3), temperatures of 170 °C, 110 °C and 140 °C were selected as the desolvation temperatures. Specifically, a high-temperature desolvation method was used, wherein the samples were placed in the oven at the corresponding temperature for 1.5 h. Then, the samples were taken for solvent removal verification by DSC, and the DSC results (Figure 7A) proved that the solvent in the multicomponent salts was completely removed. On this basis, PXRD characterization of the desolvated samples showed that all of the above multicomponent salts were converted to MET–RHE salts by high-temperature desolvation without transforming into new crystalline forms (Figure 7B). Therefore, only the follow-up evaluation of medicinal property in vitro and the study of hypoglycemic activity in vivo for MET–RHE were carried out.

### 3.6. Stability Study

**Figure 8:**
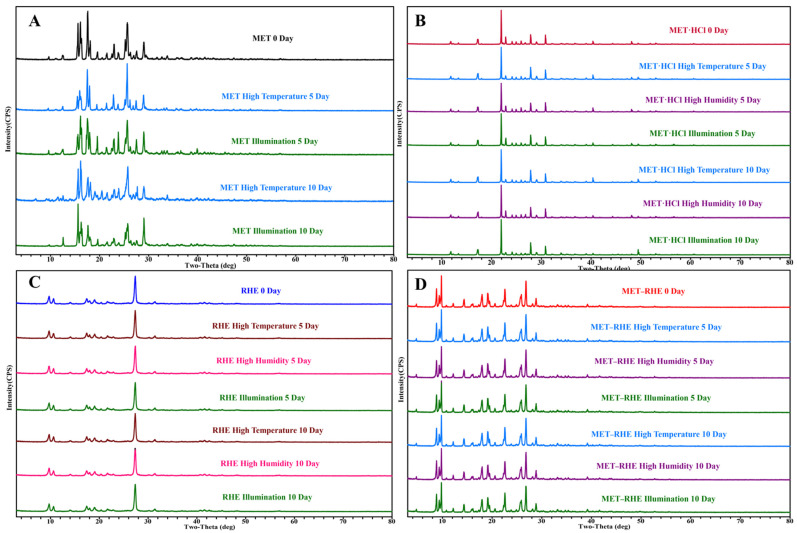
PXRD comparisons of stability study.

The stability of MET (A), MET·HCl (B), RHE (C), and MET–RHE (D) was evaluated in high-temperature, high-humidity, and illuminated environments by weighing 50 mg samples for PXRD characterization on days 0, 5 and 10, respectively (Figure 8). During the stability investigation experiments, the stability results of MET were not satisfactory, as evidenced by the significant changes in the PXRD patterns of MET samples after 10 days in a high-temperature and illuminated environment (Figure 8A). In addition, MET adsorbed water from the environment in a solution state in only 5 days under high humidity because of its hygroscopicity. In compared with MET, the stability of MET·HCl was significantly improved (Figure 8B), and it could remain stable in a high-temperature, high-humidity, and illuminated environment within 5 days. Especially, almost no change was observed in the PXRD pattern under high humidity within 10 days, indicating that MET·HCl can basically maintain stability under high humidity. However, the characteristic peaks at 2θ of 49.48° in the PXRD changed under the high-temperature and illuminated environment for 10 days, indicating that MET·HCl could not remain stable for a long time in the high-temperature and illuminated environment. Considering the good stability of RHE, its PXRD patterns of RHE did not change significantly under a high-temperature, high-humidity, and illuminated environment for 10 days. Similarly, the PXRD patterns of MET–RHE did not change significantly within 10 days in the high-temperature, high-humidity, and illuminated environment, confirming that the stability of MET was also improved significantly by forming salt with RHE, and the effect was even better than that of MET·HCl.

### 3.7. Hygroscopicity Study

**Figure 9:**
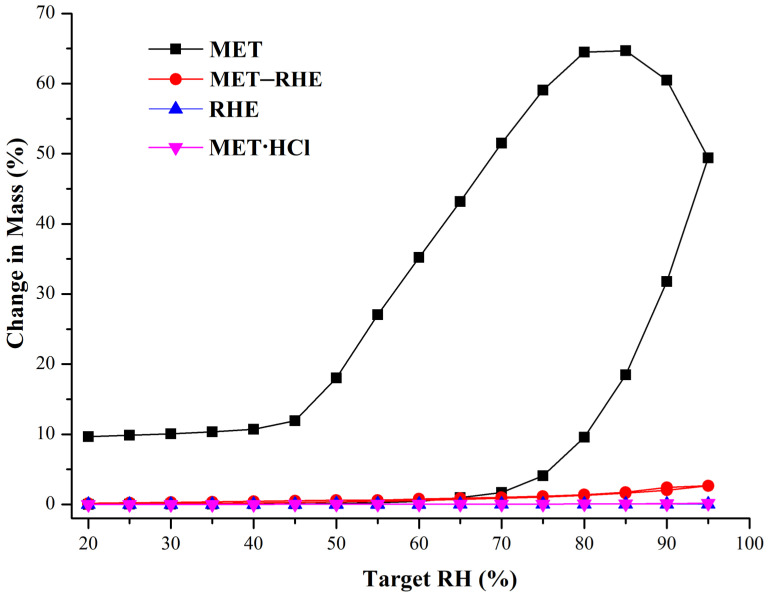
DVS comparisons of MET, RHE, MET–RHE and MET·HCl.

**Figure 10:**
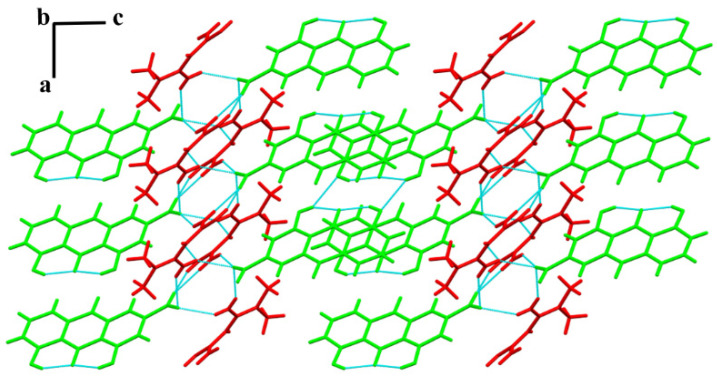
A three-dimensional (3D) structure of MET–RHE (red: MET, green: RHE).

Optimizing the hygroscopicity of MET is the main improvement target in the salification strategy of MET [28]. Therefore, the hygroscopicity of MET and MET–RHE need to be examined and compared, and the commercially available forms of MET, metformin hydrochloride (MET·HCl), and RHE need to be included. The DVS results of the above four samples are shown in Figure 9 and Table S5. In compared with MET–RHE, RHE, and MET·HCl, MET has serious hygroscopicity, wherein MET begins to absorb moisture rapidly with the increase in relative humidity. When the relative humidity reached 95%, the weight increase of MET sample reached 49.41%. Considering the extremely high hygroscopicity of MET, the MET samples did not reach equilibrium at 95% RH, even though the equilibration time was adjusted upward to 120 min. Hence, the MET samples continued to absorb moisture and gain weight with the decrease in RH. Satisfactory results were obtained when the MET–RHE sample gained only 2.66% in weight under up to 95% relative humidity, which is slightly higher than the MET·HCl and RHE sample but significantly lower than the MET sample based on the graph. This finding indicates that the salification of MET with RHE greatly optimized the hygroscopicity problem of MET, even to a level that is almost comparable to that of the commercially available form of MET (MET·HCl). After completing the DVS experiment, the samples were returned for PXRD characterization. The PXRD patterns of MET–RHE, RHE, and MET·HCl samples (Figure S2) showed little change before and after the DVS experiment, while the PXRD patterns of the MET sample obviously changed. This finding was obtained possibly because the MET still had a 9.66% weight gain after the end of the desorption cycle. From the structural point of view, in the MET–RHE crystal structure, the hydrophilic MET cations are sandwiched between hydrophobic RHE anions, forming a sandwich structure, as shown in Figure 10. Considering the above special three-dimensional structure, MET–RHE reduced the contact and interaction between MET and water molecules via physical isolation, thus greatly optimizing the hygroscopicity of MET.

### 3.8. Powder Dissolution and Solubility In Vitro

**Figure 11:**
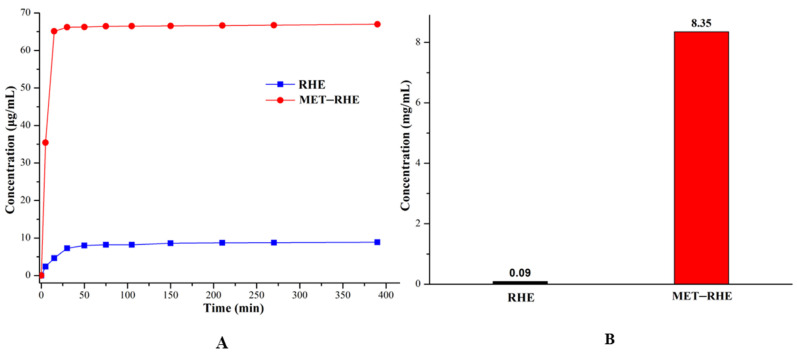
(**A**) Powder dissolution of RHE and MET–RHE. (**B**) Solubility comparisons of RHE and MET–RHE.

Considering that water solubility is one of the important factors affecting drug absorption and bioavailability [71,72], the dissolution and solubility of MET–RHE need to be evaluated. Based on the analysis and comparing the dissolution curves of RHE and MET–RHE in Figure 11A, the dissolution rate of MET–RHE (7.09 µg/mL⋅min^−1^) is much higher than that of RHE (0.48 µg/mL⋅min^−1^) based on the comparison of the slope of the first 5 min of the dissolution experiment. Satisfactorily, the in vitro solubility test (Figure 11B) showed that the equilibrium solubility of MET–RHE (8.35 mg/mL) was improved by nearly 93 times compared with RHE (0.09 mg/mL), which was very surprising, providing scientific support for improving the in vivo bioavailability of RHE. As a supplement, the characteristic peaks of the PXRD patterns (Figure S3) of MET–RHE and RHE before and after the solubility test were almost unchanged, confirming that no other new phases such as hydrate were generated during the solubility test. This finding demonstrates the reliability of the sample solubility results. The ability of MET–RHE to exhibit superior solubility compared with RHE may be related to the change of crystal structure after salification of RHE and MET. The insolubility of RHE may be related to its stable dimer structure, whereas in the three-dimensional structure of MET–RHE (Figure 10), MET is alternately arranged with RHE. Moreover, when MET–RHE came into contact with water molecules, MET with higher solubility dissolved in water first, and the remaining RHE molecules had difficulty supporting the original three-dimensional structure. This process may lead to disintegration and collapse of the original structure, thus dissolving in water. Therefore, the solubility of poorly soluble drugs can be improved via salification strategy [73,74], which can effectively optimize the level of drug absorption and improve bioavailability.

### 3.9. In Vivo Hypoglycemic Activity Evaluation

**Figure 12:**
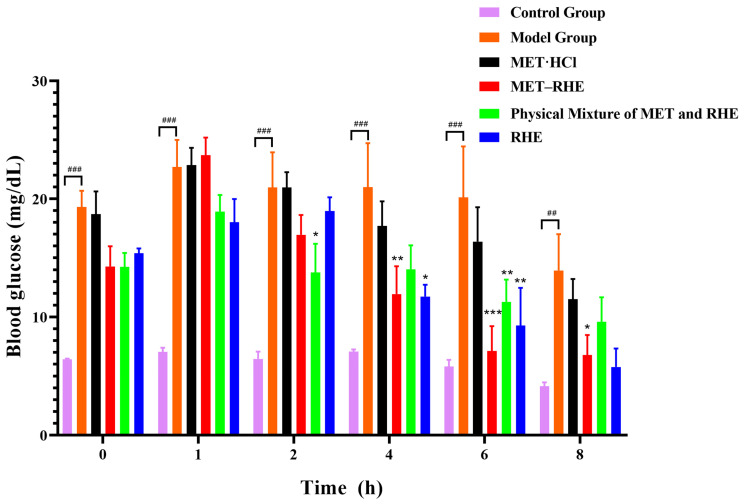
Blood glucose levels were measured at 0, 1, 2, 4, 6 and 8 h after administration. Values shown are the mean ± SEM. *n* = 3–5. ## *p* < 0.01 and ### *p* < 0.001 vs. control group. * *p* < 0.05, ** *p* < 0.01, and *** *p* < 0.001 vs. model group.

Based on the excellent physicochemical properties of MET–RHE in vitro, its hypoglycemic activity in mice was evaluated. As shown in Figure 12, the blood glucose levels in the MET–RHE group remained lower than those in the MET·HCl group for 8 h, indicating that MET and RHE enhanced hypoglycemic activity by achieving a drug combination through salification strategy. Based on the comparison of the blood glucose level within 8 h between the MET–RHE group and the physical mixture group, the physical mixture had a faster hypoglycemic rate before 4 h, while the MET–RHE group showed better hypoglycemic activity after 4 h. Therefore, the inconsistent release problem caused by the pharmacokinetic mismatch between MET and RHE may be alleviated by salt formation, which slowed down the initial period of MET rapid release, helped in reducing the stimulation of MET to the gastrointestinal tract, and prolonged the half-life of MET. In addition, after 6 h, the blood glucose level in the MET–RHE group ceased to decrease, while the RHE group still maintained a decreasing trend. Therefore, RHE has good hypoglycemic activity and has a longer half-life than MET. Notably, compared with the improvement of in vitro dissolution, the hypoglycemic activity of MET–RHE in mice did not show satisfactory optimization results, possibly because MET–RHE is a multicomponent salt with strong base and weak acid. Moreover, the strong acid environment will destroy the structure of the multicomponent salt, resulting in malabsorption. After oral administration, MET–RHE was destroyed by the strong acid environment in gastric juice, thus resulting in the circulating dose in mice [75]. In conclusion, through the optimization of combined medication, the excellent physicochemical properties in vitro are expected to translate into satisfactory pharmacological properties in vivo.

## 4. Conclusions

Optimizing the physicochemical properties of drugs, improving their bioavailability, and enhancing efficacy are some of the important topics for pharmaceutical researchers. The important contribution of this paper is the design and preparation of four multicomponent pharmaceutical salts via salification. SXRD and other methods were used to characterize structures of the salts, and the solvent removal experiment of multicomponent salt solvates was completed under PXRD monitoring. In vitro stability and solubility experiments showed that the solubility of RHE was improved by about 93 times, while the stability of MET was optimized, indicating the complementary advantages of multicomponent pharmaceutical salts. The hypoglycemic activity of MET–RHE in mice showed that MET–RHE exhibited a certain hypoglycemic advantage compared with MET·HCl and the physical mixture of raw ingredients. In addition, given that both MET and RHE exhibit some activity in alleviating diabetic nephropathy, the above studies provide a new approach to the development of new agents for the combined treatment of diabetes and diabetic complications to some extent.

## Figures and Tables

**Table 1:** Crystal data and structure refinement parameters for MET–RHE, MET–RHE–ethanol–H_2_O, and MET–RHE–acetonitrile.

Parameters	MET–RHE	MET–RHE–Ethanol–H_2_O	MET–RHE–Acetonitrile
Empirical formula	C_19_H_19_N_5_O_6_	C_21_H_27_N_5_O_8_	C_40_H_41_N_11_O_12_
Formula weight	413.39	477.47	867.84
Crystal size/mm	0.15 × 0.22 × 0.27	0.35 × 0.30 × 0.13	0.25 × 0.23 × 0.21
Description	block	block	block
Crystal system	triclinic	triclinic	monoclinic
Space group	*P-1*	*P-1*	*P21*/*c*
a (Å)	5.492 (1)	7.295 (1)	14.151 (1)
b (Å)	9.856 (1)	7.619 (1)	13.658 (1)
c (Å)	17.707 (1)	19.721 (3)	21.051 (2)
α (°)	98.989 (3)	86.327 (8)	90
β (°)	90.299 (3)	87.895 (7)	98.372 (1)
γ (°)	96.771 (4)	87.742 (6)	90
Volume (Å3)	939.9 (1)	1092.4 (2)	4025.6 (2)
Z	2	2	4
F(000)	432	504	1816
Density (g·cm^−3^)	1.461	1.452	1.432
Reflections with I > 2σ (I)	2759	3648	5645
Rindexs (I > 2σI)	R1 = 0.0926 wR2 = 0.2759	R1 = 0.0468 wR2 = 0.1091	R1 = 0.0592 wR2 = 0.1673
S	1.045	0.985	1.037

**Table 2:** The interaction energies in different types: MET–RHE, MET–RHE–ethanol–H_2_O, and MET–RHE–acetonitrile.

Interaction Style	Interaction Energy (kcal/mol)
MET–RHE ^1^	−76.54
MET–RHE ^2^	−83.27
MET–RHE ^3^	−90.19
MET–RHE–ethanol–H_2_O ^1^	−79.34
MET–RHE–ethanol–H_2_O ^2^	−79.68
MET–RHE–acetonitrile ^1^	−96.79
MET–RHE–acetonitrile ^2^	−77.85
MET–RHE–acetonitrile ^3^	−73.52

Note: The superscripts in the table represent the different types of interaction energies in multicomponent salts.

**Table 3:** The molecular planarity analysis results.

Salt	MPP	SDP	Interaction Energy (kcal/mol)	H-Bond Distance (Å)
MET–RHE	0.1642	1.1686	−4.68	2.96
MET–RHE–ethanol–H_2_O	0.1938	1.1437	−4.43	2.88
MET–RHE–acetonitrile	0.1250	0.6812	−5.23	2.85

## Data Availability

All experimental data required to reproduce the findings from this study will be made available to interested investigators.

## Supplementary Materials

The following are available online at https://www.mdpi.com/article/10.3390/pharmaceutics15041196/s1, Figure S1. TG profiles of MET–RHE, MET–RHE–H_2_O, MET–RHE–ethanol–H_2_O, MET–RHE–acetonitrile; Figure S2. The PXRD patterns of samples after DVS experiments; Figure S3. The PXRD patterns of samples after solubility tests; Table S1. Weight loss steps of solvents in the TG curves of A, B and C compared with theoretical weight loss percentage; Table S2. Detailed hydrogen bonding information of MET–RHE; Table S3. Detailed hydrogen bonding information of MET–RHE–ethanol–H_2_O; Table S4. Detailed hydrogen bonding information of MET–RHE–acetonitrile; Table S5. The DVS data of MET, MET·HCl, MET–RHE.
