# Supplementary material for: Versatile Solid Modifications of Multicomponent Pharmaceutical Salts: Novel Metformin–Rhein Salts Based on Advantage Complementary Strategy Design
Source: Pharmaceutics. 2023 Apr 9;15(4):1196. doi: 10.3390/pharmaceutics15041196 (PMC10142746; doi:10.3390/pharmaceutics15041196)
Supplement: Supplementary file 1 [file pharmaceutics-15-01196-s001.zip › pharmaceutics-2262781-supplementary.pdf]

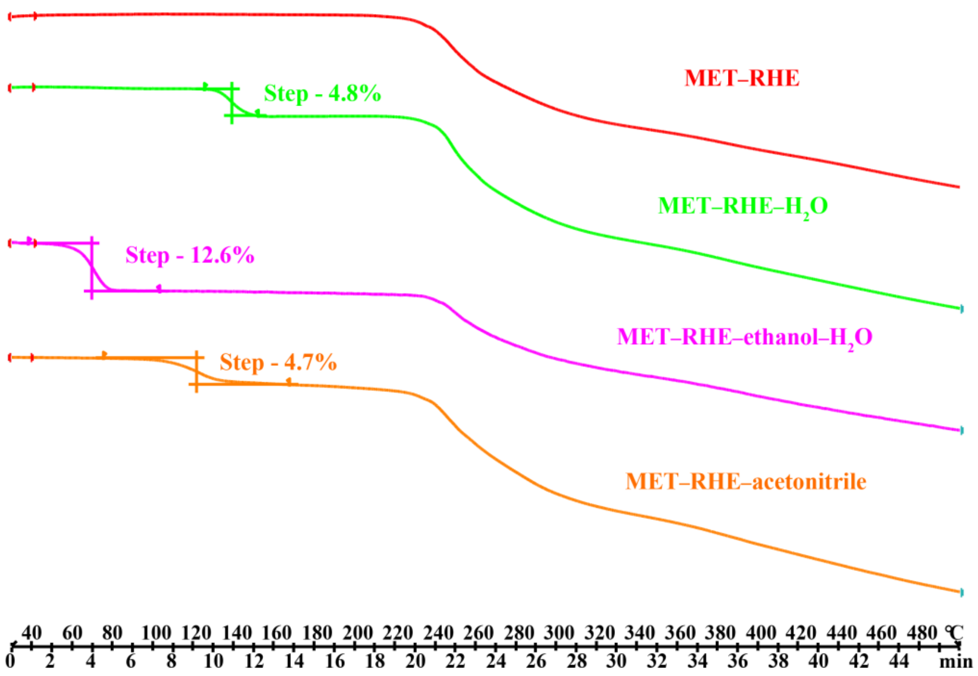


**Figure S1.** TG profiles of MET–RHE, MET–RHE–$H_2O$, MET–RHE–ethanol–$H_2O$, MET–RHE–acetonitrile.

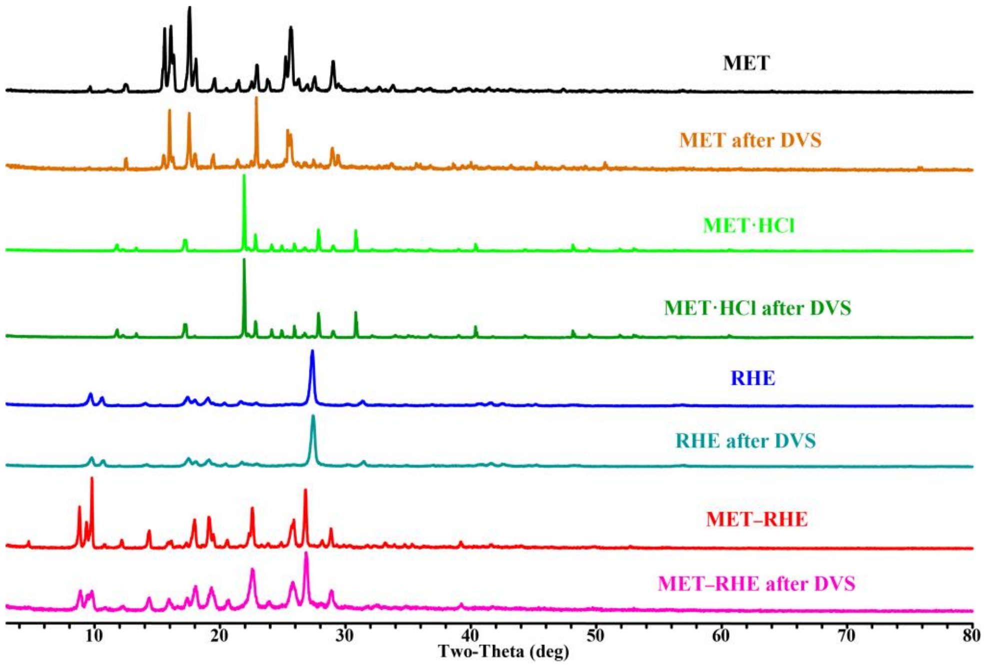


**Figure S2.** The PXRD patterns of samples after DVS experiments

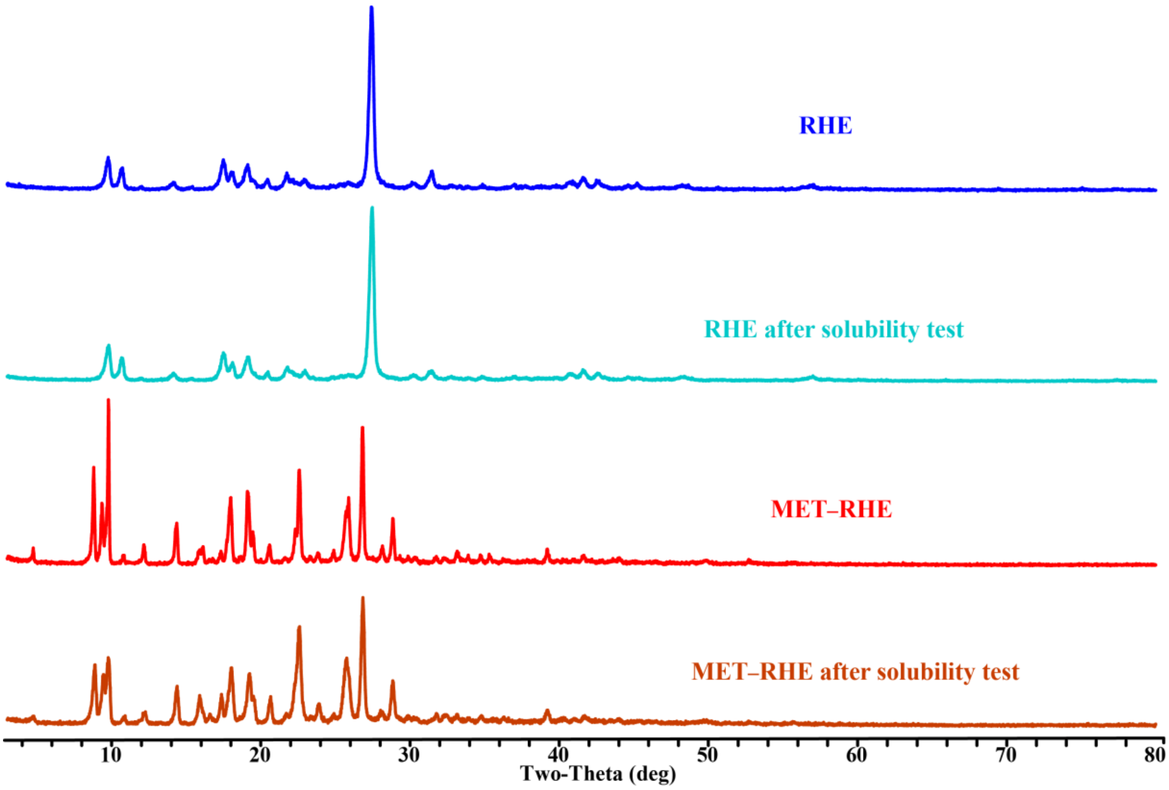


**Figure S3.** The PXRD patterns of samples after solubility tests

**Table S1.** Weight loss steps of solvents in the TG curves of A, B and C compared with theoretical weight loss percentage.

| Sample | Actual Weight Loss (%) | Theoretical Weight Loss (%) |
|---|---|---|
| MET–RHE–$H_2O$ | 4.8 | 4.2 |
| MET–RHE–ethanol–$H_2O$ | 12.6 | 13.4 |
| MET–RHE–acetonitrile | 4.7 | 4.7 |

**Table S2.** Detailed hydrogen bonding information of MET–RHE.

| D–H···A (Å) | d(D–H) (Å) | d(H···A) (Å) | d(D···A) (Å) | ∠D–H···A (deg) |
|---|---|---|---|---|
| $O_3$–$H_3$···$O_4$ | 0.82 | 1.90 | 2.60 | 143.92 |
| $O_3$–$H_3$···$O_5^a$ | 0.82 | 2.60 | 3.23 | 133.77 |
| $O_5$–$H_5$···$O_4$ | 0.82 | 1.89 | 2.61 | 145.33 |
| $O_5$–$H_5$···$O_4^b$ | 0.82 | 2.50 | 2.99 | 119.37 |
| $N_2$–$H_{2A}$···$O_2^c$ | 0.86 | 2.16 | 2.92 | 147.49 |
| $N_2$–$H_{2B}$···$O_2^d$ | 0.86 | 2.24 | 2.98 | 144.19 |
| $N_2$–$H_{2B}$···$N_4$ | 0.86 | 2.38 | 2.86 | 115.64 |
| $N_3$–$H_{3B}$···$N_5^e$ | 0.86 | 2.34 | 3.01 | 135.48 |
| $N_4$–$H_{4A}$···$O_2^f$ | 0.86 | 2.20 | 2.89 | 137.08 |
| $N_4$–$H_{4A}$···$N_2$ | 0.86 | 2.41 | 2.86 | 112.35 |
| $N_4$–$H_{4B}$···$O_1$ | 0.86 | 2.17 | 2.93 | 145.94 |
| $N_5$–$H_{5A}$···$O_1$ | 0.99 | 1.83 | 2.77 | 156.83 |

Symmetry codes: (a) -x+3, -y+1, -z (b) -x+3, -y+1, -z (c) -x, -y+1, -z+1 (d) x-1, y, z (e) -x, -y+2, -z+1 (f) -x+1, -y+1, -z+1.

**Table S3.** Detailed hydrogen bonding information of MET–RHE–ethanol–$H_2O$.

| D–H···A (Å) | d(D–H) (Å) | d(H···A) (Å) | d(D···A) (Å) | ∠D–H···A (deg) |
|---|---|---|---|---|
| $O_3$–$H_3$···$O_4$ | 0.84 | 1.86 | 2.58 | 144.02 |
| $O_3$–$H_3$···$O_4$[a] | 0.84 | 2.47 | 2.88 | 110.80 |
| $O_5$–$H_5$···$O_4$ | 0.84 | 1.85 | 2.59 | 145.13 |
| $O_5$–$H_5$···$O_3$[b] | 0.84 | 2.65 | 3.29 | 134.44 |
| $N_5$–$H_{5A}$···$O_1$ | 0.88 | 2.11 | 2.91 | 151.32 |
| $N_5$–$H_{5B}$···$N_3$[c] | 0.88 | 2.20 | 3.08 | 174.13 |
| $N_4$–$H_{4A}$···$O_7$ | 0.88 | 2.12 | 2.96 | 159.47 |
| $N_4$–$H_{4B}$···$O_7$[d] | 0.88 | 2.10 | 2.88 | 147.47 |
| $N_2$–$H_{2A}$···$O_8$[e] | 0.88 | 2.22 | 2.92 | 136.78 |
| $N_2$–$H_{2B}$···$O_1$[f] | 0.88 | 2.19 | 2.89 | 137.07 |
| $O_7$–$H_{7A}$···$O_2$ | 0.84 | 1.80 | 2.64 | 173.09 |
| $O_8$–$H_{8A}$···$O_6$ | 0.86 | 2.07 | 2.92 | 176.41 |
| $O_8$–$H_{8B}$···$O_2$[g] | 0.85 | 0.98 | 2.82 | 169.88 |

Symmetry codes: (a) -x+1, -y, -z (b) -x+1, -y, -z (c) -x+1, -y+1, -z-1 (d) -x, -y, -z-1 (e) -x,-y+1,-z-1 (f) -x,-y+1,-z-1 (g) x, y+1, z.

**Table S4.** Detailed hydrogen bonding information of MET–RHE–acetonitrile.

| D–H···A (Å) | d(D–H) (Å) | d(H···A) (Å) | d(D···A) (Å) | ∠D–H···A (deg) |
|---|---|---|---|---|
| $O_{1B}$–$H_{1B}$···$O_{4B}$ | 0.82 | 1.87 | 2.59 | 144.87 |
| $O_{1B}$–$H_{1B}$···$O_{4A}$[a] | 0.82 | 2.37 | 2.86 | 118.83 |
| $O_{1A}$–$H_{1A}$···$O_{4B}$ [b] | 0.82 | 2.36 | 2.84 | 118.38 |
| $O_{1A}$–$H_{1A}$···$O_{4A}$ | 0.82 | 1.87 | 2.58 | 145.30 |
| $O_{3B}$–$H_{3B}$···$O_{4B}$ | 0.82 | 1.89 | 2.60 | 144.37 |
| $O_{3B}$–$H_{3B}$···$O_{1A}$[c] | 0.82 | 2.48 | 3.11 | 134.85 |
| $N_{4B}$–$H_{4BA}$···$O_{5A}$[d] | 0.86 | 2.18 | 2.99 | 155.89 |
| $N_{4B}$–$H_{4BB}$···$N_{2B}$ | 0.86 | 2.47 | 2.89 | 111.31 |
| $N_{4B}$–$H_{4BB}$···$N_{1Y}$[e] | 0.86 | 2.37 | 3.06 | 137.90 |
| $N_{2B}$–$H_{2BA}$···$O_{6B}$[f] | 0.86 | 2.18 | 3.03 | 171.95 |
| $N_{2B}$–$H_{2BB}$···$O_{6B}$ | 0.86 | 2.21 | 2.90 | 137.70 |
| $N_{2B}$–$H_{2BB}$···$N_{4B}$ | 0.86 | 2.47 | 2.89 | 110.96 |
| $N_{1A}$–$H_{1AA}$···$O_{5B}$ | 0.86 | 2.29 | 3.04 | 146.16 |
| $N_{1A}$–$H_{1AB}$···$O_{6A}$[g] | 0.86 | 2.02 | 2.82 | 152.75 |
| $O_{3A}$–$H_{3A}$···$O_{1B}$[h] | 0.82 | 2.49 | 3.12 | 135.20 |
| $O_{3A}$–$H_{3A}$···$O_{4A}$ | 0.82 | 1.88 | 2.59 | 143.38 |
| $N_{1B}$–$H_{1BA}$···$O_{6A}$[i] | 0.86 | 2.06 | 2.83 | 148.75 |
| $N_{1B}$–$H_{1BB}$···$O_{5B}$[j] | 0.86 | 2.12 | 2.90 | 151.21 |
| $N_{2A}$–$H_{2AA}$···$O_{5B}$[k] | 0.86 | 2.54 | 3.18 | 132.31 |
| $N_{2A}$–$H_{2AA}$···$N_{4A}$ | 0.86 | 2.47 | 2.89 | 110.79 |
| $N_{2A}$–$H_{2AB}$···$O_{5A}$[l] | 0.86 | 2.11 | 2.95 | 167.25 |
| $N_{4A}$–$H_{4AA}$···$O_{6B}$[m] | 0.86 | 2.22 | 3.02 | 153.76 |
| $N_{4A}$–$H_{4AB}$···$O_{2A}$ | 0.86 | 2.43 | 2.89 | 113.64 |
| $N_{4A}$–$H_{4AB}$···$N_{1Y}$ | 0.86 | 2.62 | 3.19 | 125.55 |

Symmetry codes: (a) x, -y+3/2, z+1/2 (b) x, -y+3/2, z-1/2 (c) x, -y+3/2, z+1/2 (d) -x+1, y-1/2, -z+1/2 (e) -x, y-1/2, -z+1/2 (f) -x, -y+1, -z (g) x-1, y, z (h) x, -y+3/2, z-1/2 (i) -x+1, -y+1, -z (j) -x, -y+1, -z (k) -x, y+1/2, -z+1/2 (l) x-1, y, z (m) x, -y+3/2, z+1/2.

**Table S5.** The DVS data of MET, MET·HCl, MET–RHE

| Target RH (%) | Change in Mass (%) | | |
|---|---|---|---|
| | **MET** | **MET–RHE** | **MET·HCl** |
| 20 | 0.00 | 0.00 | 0.00 |
| 25 | 0.02 | 0.04 | 0.00 |
| 30 | 0.04 | 0.09 | 0.00 |
| 35 | 0.05 | 0.14 | 0.01 |
| 40 | 0.07 | 0.20 | 0.01 |
| 45 | 0.09 | 0.26 | 0.01 |
| 50 | 0.13 | 0.34 | 0.02 |
| 55 | 0.23 | 0.44 | 0.02 |
| 60 | 0.45 | 0.57 | 0.02 |
| 65 | 0.97 | 0.71 | 0.03 |
| 70 | 1.67 | 0.86 | 0.04 |
| 75 | 4.06 | 1.04 | 0.05 |
| 80 | 9.59 | 1.28 | 0.06 |
| 85 | 18.49 | 1.61 | 0.08 |
| 90 | 31.79 | 2.03 | 0.11 |
| 95 | 49.41 | 2.66 | 0.17 |
| 90 | 60.50 | 2.41 | 0.11 |
| 85 | 64.68 | 1.72 | 0.08 |
| 80 | 64.48 | 1.37 | 0.06 |
| 75 | 59.09 | 1.15 | 0.05 |
| 70 | 51.50 | 0.99 | 0.04 |
| 65 | 43.17 | 0.87 | 0.04 |
| 60 | 35.20 | 0.77 | 0.03 |
| 55 | 27.05 | 0.58 | 0.02 |
| 50 | 18.04 | 0.60 | 0.02 |
| 45 | 11.92 | 0.52 | 0.01 |
| 40 | 10.71 | 0.44 | 0.01 |
| 35 | 10.34 | 0.36 | 0.01 |
| 30 | 10.05 | 0.28 | 0.01 |
| 25 | 9.84 | 0.22 | 0.00 |
| 20 | 9.66 | 0.15 | 0.00 |
